# *Drosophila* Brakeless Interacts with Atrophin and Is Required for Tailless-Mediated Transcriptional Repression in Early Embryos 

**DOI:** 10.1371/journal.pbio.0050145

**Published:** 2007-05-15

**Authors:** Achim Haecker, Dai Qi, Tobias Lilja, Bernard Moussian, Luiz Paulo Andrioli, Stefan Luschnig, Mattias Mannervik

**Affiliations:** 1 Developmental Biology, Wenner-Gren Institute, Stockholm University, Stockholm, Sweden; 2 Abteilung Genetik, Max-Planck Institut für Entwicklungsbiologie, Tübingen, Germany; 3 Department of Genetics and Evolution, University of Sao Paulo, Sao Paulo, Brazil; Stanford University, United States of America

## Abstract

Complex gene expression patterns in animal development are generated by the interplay of transcriptional activators and repressors at *cis*-regulatory DNA modules (CRMs). How repressors work is not well understood, but often involves interactions with co-repressors. We isolated mutations in the *brakeless* gene in a screen for maternal factors affecting segmentation of the *Drosophila* embryo. Brakeless, also known as Scribbler, or Master of thickveins, is a nuclear protein of unknown function. In *brakeless* embryos, we noted an expanded expression pattern of the *Krüppel (Kr)* and *knirps (kni)* genes. We found that Tailless-mediated repression of *kni* expression is impaired in *brakeless* mutants. Tailless and Brakeless bind each other in vitro and interact genetically. Brakeless is recruited to the *Kr* and *kni* CRMs, and represses transcription when tethered to DNA. This suggests that Brakeless is a novel co-repressor. Orphan nuclear receptors of the Tailless type also interact with Atrophin co-repressors. We show that both *Drosophila* and human Brakeless and Atrophin interact in vitro, and propose that they act together as a co-repressor complex in many developmental contexts. We discuss the possibility that human Brakeless homologs may influence the toxicity of polyglutamine-expanded Atrophin-1, which causes the human neurodegenerative disease dentatorubral-pallidoluysian atrophy (DRPLA).

## Author Summary

Nuclear receptors play important roles in embryonic development and cellular differentiation by regulating gene expression at the level of transcription. The functions of transcriptional repressors, including nuclear receptors, are often mediated by other proteins, so-called co-repressors. We performed a genetic screen in the fruit fly Drosophila melanogaster to search for novel co-repressor proteins. We isolated mutations in the *brakeless* gene that alter normal transcriptional repression in early fly embryos. Brakeless was already known to regulate axon guidance in the eye, larval behavior, and gene expression in wing imaginal discs. However, the molecular function of this protein was unknown. Here we show that Brakeless is a co-repressor required for function of the Tailless nuclear receptor. Tailless was previously shown to interact with another co-repressor, Atrophin. Here, we demonstrate that Brakeless and Atrophin can bind to one another and that this interaction is conserved between a human Brakeless homolog, ZNF608, and human Atrophin-1. A polyglutamine expansion in Atrophin-1 is the cause of the neurodegenerative disease dentatorubral-pallidoluysian atrophy (DRPLA). It is possible that the interaction with ZNF608 could contribute to the pathogenesis of polyglutamine-expanded Atrophin-1.

## Abstract 3

Co-repressor activity of Brakeless with Tailless in *Drosophila* regulates segmentation genes *Krüppel* and *knirps.* Interactions between Brakeless and Atrophin may be important in developmental processes including the human neurodegenerative disease dentatorubral-pallidoluysian atrophy.

## Introduction

The generation of complex spatial and temporal gene expression patterns during embryo development is achieved through gene regulatory networks in which broadly distributed transcriptional activators act in combination with repressors with a more restricted distribution (reviewed in [1]). Repressors have an essential role in establishing gene expression boundaries. The mechanisms by which repressors act is not well understood, but may involve competition for DNA binding sites, inhibition of activator function (quenching), and direct repression (reviewed in [2–5]). Many activators and repressors require co-regulators for activity (reviewed in [6,7]). One way that co-regulators work is to modulate the chromatin structure in order to facilitate or restrict transcription initiation complex assembly. However, co-regulators may have other functions as well, such as mediating an association between transcription factors and the basal transcription machinery. It is possible that the type of co-regulator that is recruited determines the mechanism of transcriptional control at use. For example, during *Drosophila* embryo development, repressors acting over a short range recruit the CtBP co-repressor, whereas several long-range repressors interact with the co-repressor Groucho (reviewed in [8]). Yet, the mechanism by which several important transcription factors in the embryo work remains unknown. We therefore set out to isolate novel transcriptional regulators that are required for *Drosophila* embryo segmentation, and identified the Brakeless protein as a co-repressor that is required for function of the transcription factor Tailless.

Segmentation of the *Drosophila* embryo is achieved through a hierarchy of transcriptional control (reviewed in [9,10]). The maternal mRNAs *bicoid (bcd)* and *nanos (nos)* localize to the anterior and posterior poles of the embryo, respectively, from where they give rise to protein gradients in the syncytial embryo. Bcd activates transcription of the *hunchback (hb)* gene and represses translation of maternal *caudal (cad)* mRNA, whereas Nanos represses translation of maternal *hb* message (reviewed in [11]). The resulting Bcd, Hb, and Cad protein gradients act in combination to turn on expression of the first zygotic patterning genes, the gap genes, in restricted domains in the embryo. The gap gene products in turn are transcriptional repressors that regulate the next level in the hierarchy, pair-rule gene expression. Pair-rule proteins are transcription factors that control the segment-polarity genes, which in turn specify the positions of the 14 segments of the animal. The positioning of gap gene expression domains relies on interpretation of the Bcd, Hb, and Cad activator gradients and on mutual repression by the gap gene products. Positional information originating from activator and gap gene repressor gradients is integrated by *cis*-regulatory DNA modules (CRMs or enhancers, reviewed in [9]). For example, in the *knirps (kni)* CRM, binding sites for the activators Cad and Bcd are present in separate modules that are distinct from a module binding the repressors Hb, Krüppel (Kr), Giant (Gt), and Tailless (Tll) [12].

In a screen for novel maternal factors required for segmentation of the *Drosophila* embryo [13], we isolated mutations in the *brakeless (bks)* gene. Bks was previously identified as a nuclear protein with unknown function required for axonal guidance in the *Drosophila* eye [14,15], where it represses Runt expression in R2 and R5 photoreceptor cells [16]. It is also known as Scribbler, due to its behavioral locomotor phenotype in larvae [17], and as Master of thickveins (mtv) because it is important for expression of the TGF-ß receptor *thickveins* in wing imaginal disks [18]. Related sequences can be found in deuterostome genomes (echinoderms and chordates), indicating that Bks proteins may play equally important roles in other organisms.

We show here that in embryos lacking maternal *bks* function (from here on referred to as *bks* mutant embryos), the *Kr* and *kni* expression patterns expand despite the presence of the known transcriptional regulators. We find that Bks is recruited to the *Kr* and *kni* CRMs, represses transcription when bound to DNA, and functionally interacts with Tll. The Tll protein is a dedicated transcriptional repressor that belongs to the NR2 subfamily of orphan nuclear receptors [19,20]. It specifies the terminal embryonic structures by repressing transcription of genes such as *Kr* and *kni* [19]. Bks is required for Tll function, and so is Atrophin [21], the homolog of human Atrophin-1, which causes the neurodegenerative disease dentatorubral-pallidoluysian atrophy (DRPLA) when a polyglutamine stretch in the protein is expanded (reviewed in [22]). We demonstrate a direct interaction between Bks and Atrophin that is conserved between their human homologs, and propose that these proteins work together as a co-repressor complex in many developmental contexts. We discuss the possibility that this interaction may be important for both the normal and pathological function of Atrophin-1.

## Results

### Severe Segmentation Defects in Embryos Derived from *brakeless (bks)* Germline Clones

**Figure 1:**
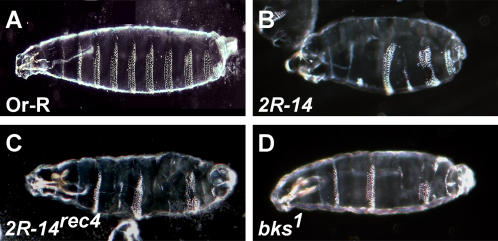
Severe Segmentation Defects in Embryos Derived from *2R-14* Germline Clones Cuticle preparations of newly hatched embryos show three thoracic (T1–T3) and eight abdominal (A1–A8) ventral denticle belts in wt embryos (A). The embryo in (B) is derived from a germline clone homozygous for the original *2R-14* mutant chromosome arm. The phenotype is intermediate to that of gap and pair-rule mutants, with several abdominal denticle belts missing. There is some variation from embryo to embryo with regard to which particular denticle belts are missing, but 100% of the embryos display a segmentation phenotype. Shown in (C) is an embryo derived from germline clones in which the *2R-14* chromosome has been cleaned by recombination. The phenotypes of these embryos are indistinguishable from those derived from the original *2R-14* chromosome. Embryos derived from *bks^1^* germline clones (D) display phenotypes virtually identical to those in *2R-14* mutant embryos.

From a screen for new maternal genes involved in embryonic pattern formation [13], we searched for mutant phenotypes that reflect defects in the transcriptional regulation of segmentation. We found a mutant, *2R-14,* that displayed severe segmentation defects in embryonic cuticle preparations (Figure 1). *2R-14* germline clone larvae show deletions of denticle belts to a variable extent. All of the abdominal segments, as well as terminal structures, can be affected, but there is no effect on dorsal-ventral patterning. Two additional alleles *(2R-278* and *2R-339)* were isolated that also show this phenotypic variability, and differ only in the frequency of the phenotypic classes. In contrast to the maternal phenotypes, patterning of *2R-14* zygotic mutant larvae is fully normal, but they die at later stages of development.

We mapped the *2R-14* locus to an approximately 600-kilobase (kb) interval between 55B and 55E, uncovered by the deficiency *Df(2R)PC4*. We performed complementation tests with all available lethal mutants in this interval and found that the *2R-14, 2R-278,* and *2R-339* alleles fail to complement the *brakeless (bks)* alleles *l(2)04440*, *bks^1^* and *bks^2^* (described in [15,17]). Thus the lethality of the *2R-14* locus maps to the *bks* gene. We cleaned the *2R-14* chromosome by recombination and did complementation tests with the *bks* alleles, as well as generated germline clone embryos from the resulting recombinants. The phenotype of these embryos is essentially identical to embryos derived from the original *2R-14* chromosome (Figure 1C). In order to confirm that mutations in the *bks* gene indeed cause a maternal segmentation defect, we recombined the independently isolated *bks^1^* and *bks^2^* alleles onto FRT^G13^ (42B) chromosomes. *bks^1^* and *bks^2^* germline clone–derived larvae and early embryos (Figure 1D and unpublished data) are phenotypically indistinguishable from *2R-14* mutants. Thus, we named our alleles *bks^14^*, *bks^278^,* and *bks^339^*.

**Figure 2:**
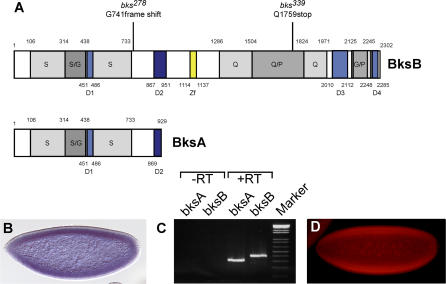
Two Bks Isoforms Are Present in Early Embryos (A) Schematic structure of the two Bks protein isoforms, Bks-A and Bks-B. The N-terminus is rich in serines and glycines, whereas the C-terminus of Bks-B is glutamine- and proline-rich. The D2 domain and the single C2H2-type zinc finger are highly conserved between insects and deuterostomes, whereas high conservation in the D1, D3, and D4 domains is limited to insects. The molecular lesions in the *2R-278* and *2R-339* alleles are indicated. (B) A wt embryo at the cellular blastoderm stage hybridized with a digoxigenin-labeled antisense *bks* probe that recognizes both *bks* isoforms. Due to the maternal contribution, *bks* transcripts are present ubiquitously in the embryo. Anterior is to the left, and dorsal is up. (C) RT-PCR experiment demonstrating the presence of both *bks-A* and *bks-B* transcripts in early embryos. RNA was isolated from 0–3–h embryos, and primers specific for bks-A or for bks-B were used in the PCR reaction. Products of the expected size were obtained after oligo-dT–primed reverse transcription, but not in the absence of reverse transcriptase. (D) A wt embryo stained with a Bks antibody raised against the D2 domain [15]. Equal staining intensity is found in all cells of the embryo. In embryos derived from *bks^278^* or *bks^14^* germline clones, nuclear staining is absent, whereas the cytoplasmic (presumably background) staining remains (unpublished data).

The *bks* locus encodes at least two proteins, Bks-A and Bks-B (Figure 2A). Bks-A is a 929–amino acid (aa)-long protein, whereas Bks-B consists of 2,302 aa and has the first 929 aa in common with Bks-A [14,15,17,18]. The only sequence similarity to known functional domains is a single C2H2 zinc finger located in the unique region of Bks-B. One additional domain (D2) is highly conserved and present also in sequences from deuterostome species (Figure S1). In vertebrates, a duplication has resulted in two genes with sequence similarity to Bks, encoding zinc-finger protein 608 (ZNF608) and ZNF609. In addition, we identified three domains (D1, D3, and D4) that are highly conserved in insects and that contain limited similarity to vertebrate sequences (Figure 2A).

We sequenced our three *bks* alleles in order to identify the molecular lesions associated with the mutations (see Figure 2A). For *bks^14^,* we were unable to amplify the first exon using various primer combinations. No mutation was detected in the rest of the gene. Sequencing of *bks^278^* revealed a 345–base pair (bp) large deletion after nucleotide 2,365 of the Bks-B cDNA. This deletion together with a 8-bp insertion causes a frame shift at aa 741 that results in addition of 79 novel amino acids. The weaker *bks^339^* allele is due to a C to T transition at position 5,485 that converts Q1758 into a stop codon. This truncates the protein before the conserved D3 domain and shows that the maternal function of the Bks-B subtype is necessary for embryo development.

### Bks Is Ubiquitously Expressed in Early Embryos

We examined the *bks* expression pattern during embryogenesis by whole-mount in situ hybridization with a probe that recognizes both *bks-A* and *bks-B*. We found *bks* mRNA to be expressed in the egg and throughout all stages of embryogenesis**.** At the blastoderm stage, ubiquitous expression is caused by the maternal contribution of the mRNA (Figure 2B). Following gastrulation, low levels of maternal transcripts remain, and *bks* is zygotically transcribed in neural cell precursors and the central nervous system (CNS) (see [17]).

In order to test whether both *bks-A* and *bks-B* are present during embryogenesis, we performed reverse transcription PCR (RT-PCR) on mRNA from early embryos (0–3 h). Using primers specific for the A and B isoforms, we detected both transcripts (Figure 2C). An antibody raised against the conserved D2 region of Bks [15] stained all cells in the embryo (Figure 2D), whereas in embryos derived from *bks^278^* or *bks^14^* germline clones, nuclear staining is absent, although we detected cytoplasmic background staining (unpublished data). In summary, we found that Bks is maternally expressed and is present ubiquitously in early embryos.

### Bks Regulates Gap Gene Expression

**Figure 3:**
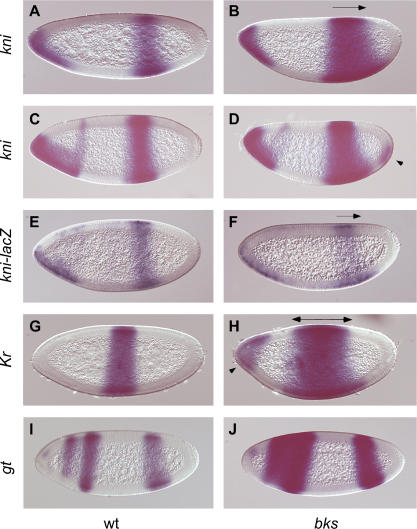
Gap Gene Expression Domains Are Expanded in *bks* Mutant Embryos Wild-type (wt) and *bks* germline clone embryos were hybridized with digoxigenin-labeled RNA probes and are oriented with anterior to the left and dorsal up. (A–D) Hybridization of a *knirps (kni)* probe to pre-cellular (A and B) and cellularizing (C and D) embryos. The *kni* pattern expands greatly towards the posterior in *bks^14^* mutant pre-cellular embryos ([B], see arrow) as compared to wt (A). In cellularizing *bks^14^* embryos (D), the *kni* pattern remains expanded compared to wt (C), and an ectopic patch occurs in the posterior-ventral part of *bks^14^* embryos (arrowhead in [D]). (E and F) A *kni-lacZ* transgene was crossed into wt (E) and *bks^278^* mutant (F) embryos, which were incubated with a *lacZ* antisense probe. Reporter gene expression expands towards the posterior in *bks^278^* mutant embryos (arrow in [F]). (G and H) Cellularizing embryos hybridized with a *Krüppel (Kr)* probe. The central domain of *Kr* expression present in wt embryos (G) expands in both an anterior and a posterior direction in *bks^14^* mutant embryos ([H], see arrow). In addition, the anterior domain (arrowhead in [H]) is expressed earlier and more broadly than in wt. (I and J) Hybridization of a *giant (gt)* probe to cellularizing embryos. Two anterior and one posterior stripe have developed at this stage in wt embryos (I). In *bks^14^* mutant embryos, the *gt* pattern is variable, but in a vast majority of embryos, there is a delay in the resolution of the anterior *gt* domain into stripes. In approximately 25% of *bks^14^* mutant embryos, the posterior stripe is reduced or even missing (unpublished data). The embryo in (J) is representative of the majority of *bks^14^* embryos at this stage.

To understand the segmentation phenotypes seen in *bks* germline clone larvae, we analyzed gene expression patterns of developmental control genes. We tested expression of all gap genes in early blastula stage embryos and found severe expression phenotypes in *bks* embryos. In wild-type (wt) pre-cellular embryos, the gap gene *kni* is expressed in two domains, one in the anterior-ventral end of the embryo, the other one as a stripe in the posterior half (Figure 3A). In *bks* mutants, this posterior domain is broadly expanded (Figure 3B). Whereas in wt, the posterior *kni* domain extends from 27% to 43% egg length (EL, where 0% is the posterior pole and 100% the anterior pole), in *bks^14^* embryos, it extends from 15% to 41% EL. In cellularizing *bks* embryos, the posterior domain remains expanded, and additional ectopic expression is found in the posterior-ventral end of the embryo (arrowhead in Figure 3D). To determine whether the effect on *kni* expression is transcriptional or post-transcriptional, we introduced a *kni* 4.4-kb CRM-*lacZ* transgene [23] into *bks* germline clone embryos. As shown in Figure 3F, *lacZ* expression expands towards the posterior as compared to wt embryos (Figure 3E). The *kni-lacZ* pattern extends from 31% to 43% EL in wt embryos, and expands to 22%–41% EL in *bks^278^* mutant embryos. We conclude that expansion of the *kni* pattern in *bks* mutant embryos is due to transcriptional deregulation.

*Kr* is first expressed in a central domain (CD) of the embryo (Figure 3G). Later, additional anterior and posterior domains are detectable (unpublished data). In *bks* embryos, the CD is broadly expanded both in an anterior and a posterior direction (Figure 3H). We measured the CD to 31%–62% EL in *bks^14^* embryos, compared to 40%–57% EL in wt. Intensity of expression also appears enhanced and persists into later stages of embryogenesis. In addition, expression of the anterior domain is enhanced, expanded, and expressed earlier in *bks* embryos as compared to wt (arrowhead in Figure 3H). Thus, Bks is necessary to restrict *Kr* expression.

The *gt* expression pattern develops from a broad domain in the anterior half and one narrower domain in the posterior half in the early blastula embryo, to three anterior stripes and a posterior stripe in cellularizing embryos at mid-cycle 14. In *bks* embryos, *gt* expression is variable, but in a majority of embryos, resolution of the anterior domain into stripes is delayed (compare Figure 3J with 3I). The posterior domain is less affected, but in about 25% of the embryos, its expression is reduced (unpublished data).

The gap genes are activated by the maternal factors Bcd, Cad, and Hb. The terminal gene products Tll and Huckebein (Hkb) act as repressors that restrict gap gene expression together with mutual inhibition by gap gene products. Expression of these upstream regulators is mostly normal in *bks* mutants (Figure S2), and cannot be responsible for the gap gene phenotypes observed.

In conclusion, three gap genes are de-repressed in *bks* mutants. Similar to previous findings [16,18], absence of Bks leads to de-repression of transcription, indicating that Bks may normally be involved in transcriptional repression.

### Tll Function Is Impaired in *bks* Embryos

Gap gene expression boundaries are set by repressor proteins. For example, Kr and Gt restrict each other's expression [24–27], and Tll represses *Kr* and *kni* [23,28–30]*.* We therefore tested whether Bks is a co-repressor required for the activity of regulators of *Kr* and *kni* expression.

**Figure 4:**
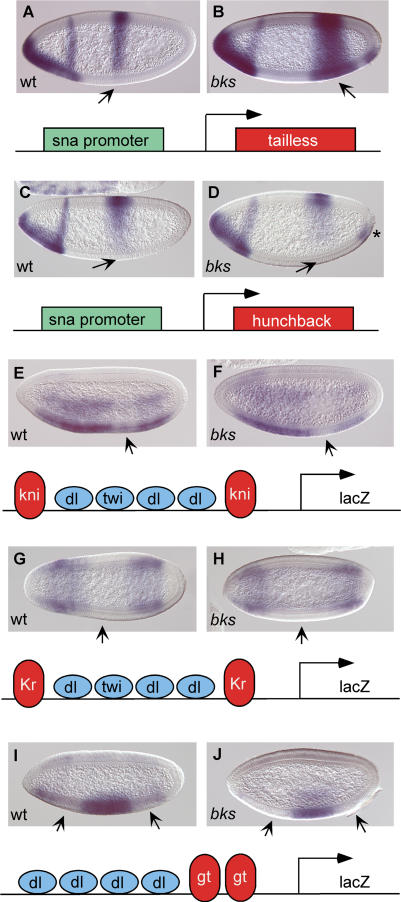
Tailless (Tll) Repressor Function Is Impaired in *bks* Embryos (A–D) Effects of ectopically expressed Tailless (Tll) and Hunchback (Hb) proteins on *kni* expression in *bks* mutant embryos. Schematic drawings of the transgenes used to drive ectopic Tll and Hb expression are depicted below the embryo images. (A and B) A *snail* promoter transgene driving Tll expression in ventral cells was crossed with wt flies or flies containing *bks^278^* germline clones. Expression of *tll* and *kni* was visualized in cellularizing embryos by fluorescent in situ hybridization (unpublished data), and by immunohistochemical detection of a digoxigenin-labeled probe, respectively. (A) A wt embryo containing the *snail-tll* transgene. The posterior *kni* stripe is repressed in ventral cells (arrow). (B) The posterior *kni* stripe is not repressed ventrally in a *bks* mutant embryo containing the *sna-tll* transgene (arrow). This shows that the repressor activity of ectopic Tll is impaired in *bks* mutants. (C and D) A *hb* transgene driven by the *snail* promoter was introduced into wt embryos or *bks^14^* germline clone embryos. Lateral views of late cellularizing embryos show that ectopic Hb can repress *kni* expression ventrally in both wt (C) and *bks* (D) mutants (arrows). Note that the posterior patch of *kni* expression that occurs in *bks* mutants is unaffected (star in [D]), presumably because the *snail* expression pattern does not extend all the way to the posterior. (E–J) Assay of endogenous Knirps (Kni), Krüppel (Kr), and Giant (Gt) function on reporter transgenes containing synthetic repressor binding sites (schematic drawings of the transgenes are presented below the embryo images). (E and F) Males harboring a *lacZ* reporter transgene driven by a modified *rhomboid* NEE enhancer with synthetic Kni binding sites were crossed with wt females or females containing *bks^14^* germline clones. Embryos were collected and hybridized with a *lacZ* probe. Ventro-lateral views of cellularized wt (E) and *bks* (F) embryos demonstrate that endogenous Kni protein represses reporter gene expression in both genotypes (arrows). (G and H) Introduction of a modified NEE reporter gene with synthetic Kr binding sites into wt embryos (G) and embryos derived from *bks^14^* germline clones (H). Ventral views of cellularized embryos hybridized with a *lacZ* antisense probe show that endogenous Kr protein can repress reporter gene expression in both genotypes (arrows). (I and J) Lateral views of a cellularized wt embryo (I) and a cellularized embryo derived from a *bks^14^* germline clone (J) containing a reporter gene with synthetic Gt binding sites, activated by a *twist* PE enhancer and the *rhomboid* NEE enhancer, stained with a *lacZ* probe. Endogenous Gt protein can repress the reporter in both wt and *bks* mutant embryos (arrows). Dorsal (dl) and Twist (twi) activators bind the *rhomboid* and *twist* enhancers.

We first investigated whether the activities of Tll and Hb are affected in *bks* embryos by misexpressing them in wt and *bks* mutant backgrounds, and compared their ability to repress transcription. We used a *snail* promoter construct that directs ectopic Tll or Hb expression in the ventral domain of the embryo (described in Protocol S1 and in [31]). When Tll is misexpressed in wt embryos, *kni* expression becomes repressed in ventral cells (Figure 4A, arrow). By contrast, in a majority of *bks* mutant embryos, the *kni* expression pattern is unaffected by misexpressed Tll (Figure 4B, arrow; and Table S1), suggesting that full Tll activity depends on wt Bks function.

On the other hand, misexpression of Hb from the *snail* promoter causes repression of *kni* in the ventral half of the embryo in both wt (Figure 4C) and in a *bks* mutant background (Figure 4D). Despite the enhanced and expanded levels of *kni* expression in *bks* embryos, ectopic Hb is sufficient to repress *kni* ventrally. The ectopic patch of *kni* expression in the posterior-ventral part of the embryo remains unaffected (star in Figure 4D), presumably because *snail* expression does not extend to the very posterior of the embryo [32].

To examine the repressor activities of Kni, Kr, and Gt proteins, we introduced *lacZ* reporter gene constructs into *bks* mutant embryos. *LacZ* expression is driven by a modified *rhomboid* neuroectoderm enhancer (NEE) that is activated on the ventral side of the embryo by the Dorsal and Twist proteins. In addition, the enhancer constructs contain either Kni, Kr, or Gt binding sites (described in [33–35]). In wt embryos, binding of the corresponding gap protein leads to repression of the reporter gene in the domain of gap gene expression (Figure 4E, 4G, and 4I). Similarly, *lacZ* expression is repressed in the gap gene expression domains in a *bks* background (Figure 4F, 4H, and 4J). Thus, in *bks* mutants, the three gap proteins Kni, Kr, and Gt are able to perform repression at least on the artificial enhancer constructs used, indicating that Bks is not required for the repressor activities of these proteins. We conclude that Tll-mediated repression is impaired in a *bks* mutant background, whereas the Hb, Kni, Kr, and Gt repressors are not affected under these conditions.

### Interactions among Bks, Tll, and Atrophin

**Figure 5:**
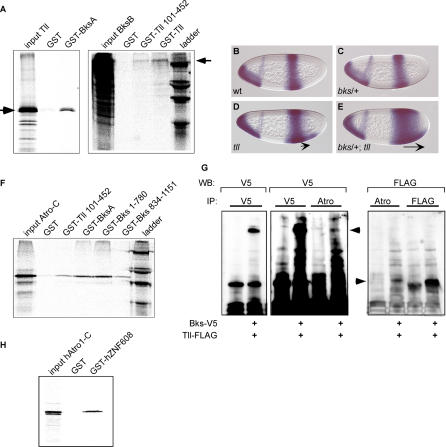
Bks Interacts with Tll and Atrophin (A) Binding of Bks to Tll in vitro. Left panel shows that in vitro–translated Tll interacts with bacterially produced GST-BksA, but not with GST alone. In the right panel, in vitro–translated Bks-B binds weakly to a GST-Tll fusion protein lacking the DNA binding domain (GST-Tll 101–452), and more strongly with GST-full-length Tll. (B–E) Genetic interaction of *bks* with *tll* mutants. Cellularizing embryos hybridized with a *kni* probe are oriented with anterior to the left and dorsal up. The *kni* pattern in wild-type (wt) embryos (B) and embryos from *bks^278^* heterozygous mothers (C) are indistinguishable. In *tll^1^* homozygous embryos (D), the posterior *kni* domain expands slightly towards the posterior. In *tll^1^* embryos derived from *bks^278^* heterozygous females (E), there is a further expansion of the *kni* pattern (see arrow). (F) Bks interacts with the C-terminus of Atrophin. Amino acids (aa) 1,324–1,966 of Atrophin binds the ligand binding domain of Tll, as well as GST-BksA. Truncation of the conserved Bks D2 region (GST-Bks 1–780) does not disrupt binding, but a weaker, independent interaction is found with the D2 domain together with the zinc finger (GST-Bks 834–1,151). (G) Bks and Tll can be co-immunoprecipitated with Atrophin from *Drosophila* S2 cells. A stable cell line expressing V5-tagged Bks-B was generated and transiently transfected with FLAG-tagged Tll. Immunoprecipitations with V5, Atrophin, and FLAG antibodies were performed from these cells and compared to normal S2 cells lacking tagged Bks and Tll. The leftmost panel shows a short exposure of a membrane immunoblotted with the V5 antibody, demonstrating the presence of Bks-V5 in transfected cells. The middle panel shows a longer exposure of the same membrane, where Bks-V5 is co-immunoprecipitated with endogenous Atrophin. In the right panel, FLAG-Tll is detected both in the Atrophin and FLAG immunoprecipitates. Arrowheads point to Bks-V5 and Tll-FLAG. (H) The human Bks homolog ZNF608 (aa 1–600) interacts with aa 600-1191 from human Atrophin-1, showing that the Bks-Atrophin interaction is evolutionarily conserved.

We tested whether the dependence of Tll repressor function on Bks might be due to a molecular interaction between these proteins. Tll and Bks-A were expressed as GST-fusion proteins in bacteria, and mixed with radiolabeled in vitro–translated proteins. As shown in Figure 5A, in vitro–translated Tll interacts with GST-BksA, and in vitro–translated Bks-B interacts more strongly with full-length Tll than with a GST fusion lacking the DNA binding domain. This shows that Bks and Tll interact in vitro, and that the Tll DNA binding domain is important for the interaction.

A functional interaction between Tll and Bks was demonstrated in vivo by genetic means. We found that lowering the dose of *bks* in a *tll* mutant background causes enhanced de-repression of *kni* expression. In embryos derived from a *tll* hypomorph, *kni* expression expands towards the posterior (compare Figure 5D with Figure 5B). By contrast, embryos receiving half the dose of maternal *bks* have an essentially wt *kni* expression pattern (Figure 5C). However, in *tll* mutant embryos with reduced amounts of maternal *bks* product, the *kni* expression pattern expands even further to the posterior (Figure 5E). These results suggest that Bks and Tll cooperate to set the normal posterior boundary of *kni* expression.

It was recently demonstrated that Tll also interacts with the Atrophin protein, and that Atrophin and Bks genetically interact in adult flies [21,36]. We therefore performed a GST pulldown assay to investigate whether Bks and Atrophin can interact in vitro. As previously published [21], the C-terminus of Atrophin interacts with the ligand binding domain of Tll (Figure 5F). We found that the Atrophin C-terminus interacts with GST-BksA as well. A truncated Bks protein (Bks 1–780) lacking the evolutionarily conserved D2 region still binds to Atrophin, but a weaker, independent interaction was also found with a Bks portion consisting of the conserved D2 region and the zinc finger (Bks 834–1,151, Figure 5F). Thus, Atrophin can bind to at least two separate parts of the Bks protein. These results show that Tll can interact with both Bks and Atrophin, and that Bks and Atrophin can bind to one another as well. This suggests that a tripartite complex consisting of Tll, Bks, and Atrophin might form. We confirmed the interactions among Bks, Atrophin, and Tll in S2 cells expressing V5-tagged Bks-B and FLAG-tagged Tll proteins. Using an Atrophin antibody, we could co-immunoprecipitate V5-tagged Bks and FLAG-tagged Tll with endogenous Atrophin (Figure 5G).

We then tested whether this interaction is evolutionarily conserved. We made a GST-fusion protein consisting of the first 600 aa of the human Bks homolog ZNF608 (including the conserved D2 domain and the zinc finger), and mixed it with radiolabeled C-terminus of human Atrophin-1. A strong interaction between these proteins was observed (Figure 5H). We conclude that the interaction between Bks and Atrophin has been conserved during evolution.

### Bks Associates with *kni* and *Kr* CRMs

**Figure 6:**
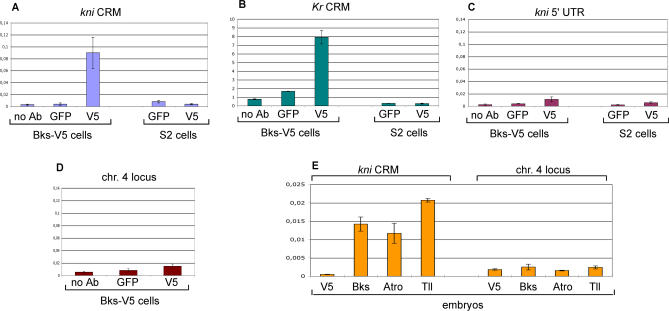
Bks Associates with the *kni* and *Kr* CRMs (A–D) Chromatin immunoprecipitations (ChIP) were performed on S2 cells or S2 cells expressing V5-tagged Bks-B protein, and associated DNA was quantified by real-time PCR. Mock immunoprecipitation (no Ab), and immunoprecipitation with a negative control antibody (GFP) and with an antibody recognizing the V5 tag (V5) were compared. PCR was performed in triplicate and compared to a standard curve of input DNA. The standard deviation is indicated. (A) An amplicon from the *kni* CRM is enriched by the V5 antibody in extract from Bks-V5–expressing cells, but not in extract from S2 cells lacking Bks-V5. (B) The V5 antibody precipitates more of *Kr* CRM DNA from Bks-V5 cells than the control antibody. No enrichment is observed in cells without Bks-V5. (C and D) Bks binding to the *kni* 5′ UTR (C) or to a locus on Chromosome 4 (D) is similar to the negative controls. (E) Bks is associated with the *kni* CRM in early embryos. ChIP followed by real-time PCR was performed on extract from 2–4-h-old embryos with negative control antibody (V5), which was compared to Bks, Atrophin, and Tll antibodies. The Bks, Atrophin, and Tll antibodies precipitated more *kni* CRM DNA than the V5 control, but similar amounts to V5 of the Chromosome 4 locus.

An interaction with Tll is expected to bring Bks to the *kni* and *Kr* CRMs to directly regulate their expression. To determine if Bks is associated with the *Kr* and *kni* CRMs, we performed chromatin immunoprecipitations (ChIP) from S2 cells expressing V5-tagged Bks-B protein. We found a 23-fold and 4.7-fold enrichment at the *kni* and *Kr* CRMs, respectively, with the V5 antibody compared to a control green fluorescent protein (GFP) antibody (Figure 6A and 6B). As a control, we performed ChIP from normal S2 cells lacking the tagged Bks protein. From these cells, the V5 antibody precipitated less *kni* and *Kr* CRM DNA than the control GFP antibody (Figure 6A and 6B). A comparable amount of *kni* 5′ UTR DNA was precipitated with the V5 antibody from V5-tagged Bks-B–expressing cells as from normal S2 cells (2.8-fold and 2.7-fold compared to GFP antibody; Figure 6C). A locus on Chromosome 4 was precipitated at a similar efficiency with V5 and GFP antibodies (1.7-fold enrichment; Figure 6D). From these results, we conclude that Bks specifically associates with *kni* and *Kr* CRM sequences when expressed in S2 cells.

We extended these results to *Drosophila* embryos using an affinity-purified antibody raised against Bks amino acids 450–620. We found an enrichment of *kni* CRM sequences with Bks, Atrophin, and Tll antibodies compared to the control V5 antibody with chromatin prepared from wt embryos (Figure 6E). No enrichment at the Chromosome 4 locus was observed (Figure 6E). In summary, both Bks and Atrophin are recruited to a Tll-regulated target gene in vivo.

### Bks Proteins Can Repress Transcription When Tethered to DNA

**Figure 7:**
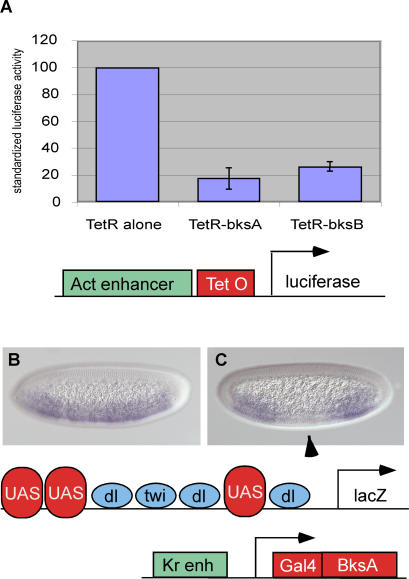
Bks Is Capable of Repressing Transcription When Tethered to DNA (A) The tetracycline repressor DNA binding domain (TetR) was fused to the coding region of bks-A or bks-B. These plasmids were co-transfected with a *luciferase* reporter gene driven by the *actin 5C* enhancer (Act enhancer) that contains tet operators (Tet O), as well as an *actin 5C*-driven *lacZ* gene to control for transfection efficiency, into mbn-2 cells. Luciferase activity (normalized for ß-galactosidase activity) of unfused TetR is set to 100%, and normalized luciferase activity of TetR-bks fusions plotted relative to the TetR. A schematic drawing of the reporter plasmid is depicted below the histogram. (B and C) Ventro-lateral view of transgenic embryos expressing *lacZ* under control of a modified *rhomboid* NEE enhancer into which Gal4 upstream activating sequences (UAS) have been inserted. *LacZ* is expressed uniformly in ventral cells in embryos only containing the reporter gene (B). In embryos that additionally express a Gal4 DNA binding domain–BksA fusion protein under control of the *Kr* CD enhancer (Kr enh), *lacZ* expression is repressed in the central, *Kr* expressing domain (C). Schematic drawings of the reporter gene and the Gal4-BksA expressing transgene are shown underneath the embryo images. Dorsal (dl) and Twist (twi) activators bind the modified *rhomboid* NEE.

Since *bks* genetically behaves as a repressor, we tested whether Bks proteins are capable of repressing transcription when tethered to a promoter. We fused *bks* coding regions to the DNA binding domain of the tetracycline repressor (TetR-DBD) and expressed the fusion constructs in *Drosophila* tissue-culture cells. We co-transfected a luciferase reporter construct driven by the *actin5C* enhancer that also contains tet operators, binding sites for the TetR-DBD (described in [37]). We compared luciferase activity of cells that expressed TetR-Bks fusion proteins with those that expressed the TetR-DBD protein alone. We found that both Bks-A and Bks-B are able to repress transcription when tethered to DNA in mbn-2 as well as in S2 cells (Figure 7A and unpublished data).

We also investigated Bks repressor activity in a transgenic embryo assay. Bks-A coding sequence was fused to the Gal4 DNA binding domain and placed under control of the *Kr* CD enhancer, which directs expression in the CD of the early embryo. A *lacZ* reporter gene containing a modified *rhomboid* NEE lacking Snail repressor sites and containing three upstream activation sequence (UAS) sites was used to monitor Gal4-Bks repressor activity (described in [34]). In a wt background, the reporter gene is expressed in ventral regions of the embryo (Figure 7B). However, when crossed into transgenic embryos expressing the Gal4-BksA fusion protein, the reporter is repressed in central regions (Figure 7C).

In conclusion, our data show that Bks proteins are capable of repressing transcription when bound to a promoter. Taken together with our other results, we conclude that Bks acts as a transcriptional co-repressor.

## Discussion

Repression plays a pivotal role in establishing correct gene expression patterns that is necessary for cell fate specification during embryo development. For example, in the early *Drosophila* embryo, repression by gap and pair-rule proteins is essential for specifying the positions of the 14 segments of the animal. The mechanisms by which transcriptional repressors delimit gene expression borders are not well understood. However, many repressors require co-repressors for function. In the *Drosophila* embryo, the CtBP and Groucho co-repressors are required for activity of many repressors (reviewed in [8,38]). More recently, Atrophin has been identified as a co-repressor for Even-skipped and Tll [21,39]. Still, co-regulators for several important transcription factors in the early embryo have not yet been identified. We therefore performed a screen for novel maternal factors that are required for establishing correct gene expression patterns in the early embryo.

From this screen, we identified mutations in the *bks* gene that cause severe phenotypes on gap gene expression and embryo segmentation. The Bks protein is evolutionarily conserved between insects and deuterostomes, but has not been characterized in any species except *Drosophila,* in which it has been shown to repress *runt* expression in photoreceptor cells and *thickveins* expression in wing imaginal disks [16,18]. However, the molecular function of Bks was unknown. We show here that Bks interacts with the transcriptional repressor Tll, is recruited to target gene CRMs, and will repress transcription when targeted to DNA.

Tll was recently shown to utilize Atrophin as a co-repressor [21]. Atrophin genetically interacts with Tll and physically interacts with its ligand binding domain. Atrophin binding is conserved in nuclear receptors within the same subfamily, such as Seven-Up in *Drosophila* as well as Tlx and COUP-TF in mammals [21,40]. When expressed in mammalian cells, *Drosophila* Atrophin and mouse Atrophin-2 interact with the histone deacetylases HDAC1 and HDAC2 [21,41]. Histone deacetylation may therefore be part of the mechanism by which Atrophin functions as a co-repressor. Another recent report described genetic interactions among *bks* and *atrophin* mutants in the formation of interocellar bristles in adult flies [36]. Furthermore, it was shown that *atrophin* mutants have virtually identical phenotypes as *bks* mutants, including de-repression of *runt* expression in the eye, *thickveins* expression in the wing, and *Kr* and *kni* expression in the embryo [21,36,42].

We now show that both proteins are recruited to the *kni* CRM, a Tll-regulated target gene, in the embryo. Importantly, we further demonstrate that Atrophin and Bks interact in vitro and that they can be co-immunoprecipitated from S2 cells. We propose that Bks and Atrophin function together as a co-repressor complex, and based on the similar *bks* and *atrophin* mutant phenotypes at several developmental stages, the complex may function throughout development. Our results are compatible with the existence of a tripartite complex consisting of Tll, Bks, and Atrophin. Bks binding to Tll is enhanced by the Tll DNA binding domain, whereas the interaction of Tll with Atrophin is mediated through the C-terminal ligand binding domain. Tll may therefore simultaneously interact with Bks and Atrophin. Alternatively, Tll interacts separately with Bks and Atrophin on the *kni* CRM. In either case, both Bks and Atrophin are required for full Tll activity. However, at high enough Tll concentration, Bks activity is dispensable. Some *bks* embryos misexpressing Tll still repress *kni* expression (Table S1), and overexpressing Tll from a heat-shock promoter can repress the posterior *kni* stripe in both wt and *bks* mutant embryos (unpublished data). For this reason, we believe that Bks and Atrophin are cooperating as Tll co-repressors, so that Tll function is only partially impaired by the absence of either one. We found that Tet-Bks–mediated repression in cells is insensitive to the deacetylase inhibitor trichostatin A (TSA; unpublished data). It is possible, therefore, that whereas Atrophin-mediated repression may involve histone deacetylation, Bks could repress transcription through a separate mechanism.

Our results have not revealed any differences between the molecular functions of the two Bks isoforms. Both Bks-A and Bks-B repress transcription when tethered to DNA, and the sequences that mediated binding to Tll and Atrophin are shared between the two isoforms. However, the *bks^339^* allele that selectively affects the Bks-B isoform causes a weaker, but comparable phenotype to the stronger *bks* alleles that disrupt both isoforms. Therefore, the C-terminus of Bks-B provides a function that is indispensable for embryo development and regulation of *kni* expression. This part of Bks-B contains two regions (D3 and D4) that are highly conserved in insects and loosely conserved in deuterostome Bks sequences, but does not resemble any sequence with known function. The only sequence similarity to domains found in other proteins is a single zinc-finger motif in Bks-B. Preliminary results indicate that the zinc finger in isolation or together with the conserved D2 domain does not exhibit sequence-specific DNA binding activity (unpublished data). Indeed, multiple zinc fingers are generally required to achieve DNA binding specificity (reviewed in [43]). Instead, Bks is likely brought to DNA through interactions with Tll and other transcription factors.

Atrophins are required for embryo development in *Caenorhabditis elegans, Drosophila,* zebrafish, and mice [39,41,42,44–47]. In vertebrates, two atrophin genes are present. Atrophin-1 is dispensable for embryonic development in mice, and lacks the N-terminal MTA-2 homologous domain that interacts with histone deacetylases [48]. However, the homologous C-termini of Atrophin-1 and Atrophin-2 can interact, and we found that this domain can also bind to the human Bks homolog ZNF608 (Figure 5H). Atrophin-1 interacts with another co-repressor–associated protein as well, ETO/MTG8, and can repress transcription when tethered to DNA [49]. These data are consistent with the emerging view that deregulated transcription may be an important mechanism for the pathogenesis of polyglutamine diseases (reviewed in [50,51]). Recent evidence indicates that interactions with the normal binding partners may cause toxicity of polyglutamine-expanded proteins such as Ataxin-1 [52]. It will be interesting to investigate whether the interaction between human Bks homologs and Atrophin-1 is important for the neuronal toxicity of polyglutamine-expanded Atrophin-1.

## Materials and Methods

Generation of germline clones, cuticle preparations, in situ hybridization and immunohistochemistry, molecular cloning, P element transformation, GST pulldowns, RT-PCR, cell culture and transient transfections, immunoprecipitation, and chromatin immunoprecipitation are described in Protocol S1.

### Bks alleles.

The *bks* alleles *bks^14^, bks^278^,* and *bks^339^* were generated on an FRT^2R-G13^–containing chromosome in germline clone ethylmethane sulfonate (EMS) screens performed in Tübingen ([13] and N. Vogt, unpublished data). Recombination mapping placed the *2R-14* locus on chromosome arm 2R between the markers *curved* (52D) and *plexus* (58E). Complementation tests with deficiencies covering this area narrowed the *2R-14* locus down to approximately 600 kb between 55B and 55E, uncovered by the deficiency *Df(2R)PC4*. We performed complementation tests with all available lethal mutants in this interval and found that the *2R-14, 2R-278,* and *2R-339* alleles fail to complement the *bks* alleles *l(2)04440*, *bks^1^,* and *bks^2^*. *l(2)04440* is a P element insertion described in [17]. The *bks^1^* and *bks^2^* EMS-induced alleles, kindly provided by Barry Dickson (described in [15]), were recombined to an FRT^2R-G13^–containing chromosome (using stock #1958 in [53]). The *bks^14^* and *bks^278^* alleles were outcrossed against an FRT^2R-G13^ c px sp/CyO hs-hid chromosome to clean the stock from additional mutations. Four different recombinants (two from both sides of the *bks* locus) were tested and showed no significant phenotypic differences from the parental chromosomes.

The *bks^14^, bks^278^,* and *bks^339^* alleles were balanced over CyO tubulin-GFP to enable isolation of homozygous mutant larvae. Genomic DNA was prepared and *bks* exonic sequences amplified by PCR, sequenced, and compared to an FRT^2R-G13^ chromosome derived from another mutant from the screen, *2R-91*.

### Genetics.

Females harboring *bks* germline clones were crossed with transgenic males to introduce various transgenes into *bks* mutant embryos. To determine if *kni* expansion in *bks* germline clone embryos is due to transcriptional control, we crossed males containing a *lacZ* reporter regulated by a 4.4-kb *kni* enhancer (GO125 *kni*4.4*lacZ,* [23]) to *bks^278^* germline clone females and analyzed the resulting embryos for *lacZ* expression using in situ hybridization.

To analyze the activity of Tll and Hb in a *bks* mutant background, males containing transgenes misexpressing *tll* or *hb* were crossed to *bks^278^* or *bks^14^* germline clone females or to wt females. Expression in a ventral domain of the embryo was achieved by use of the *snail* promoter (*sna:tll* stocks x196 and x197, described in Protocol S1, and *sna:hb* stock x227, described in [31]). The constructs contain transcriptional stop signals flanked by FRT sites downstream of the *sna* promoter to allow maintenance of transgenic lines. Ventral expression was activated by crossing in a ß*2-tubulin-FLP* transgene [54]. Male progeny containing both *FLP* and *sna* promoter transgenes (in whose spermatocytes recombination occurred) were crossed to virgins with *bks* germline clones or to wt virgins; embryos were then collected and processed for in situ hybridization with a *kni* probe.

To test the repressor activities of Kni, Kr, and Gt proteins in a *bks* germline clone background, we crossed wt females or females with *bks^14^* germline clones with males containing modified *rhomboid* NEE enhancers. The *NEE-kni-lacZ* transgene (lab stock A45) is described in [33] and contains synthetic Kni binding sites, but lacks Snail sites. *NEE-Kr-lacZ* (lab stock G5.5) contains synthetic Kr sites, but lacks Snail sites, and is described in [34]. The 2x*gt*-55 *lacZ* reporter gene is described in [35]. It is activated by the *rhomboid* NEE as well as the 2xPE *twist* enhancer and contains two Gt sites situated 55 bp upstream of the transcription start site. Embryos were collected and fixed 2–4 h after egg laying, and *lacZ* expression patterns were analyzed by in situ hybridization.

Flies containing a modified *rhomboid* NEE-*lacZ* reporter gene with three UAS sites (described in [34]) were crossed to wt or Kreggy-BksA transgenic flies (see Protocol S1), embryos collected, and *lacZ* reporter gene expression analyzed by in situ hybridization.

Genetic interactions between *bks^278^* and *tll^1^* were tested by crossing *bks^278^*/+; *tll^1^*/+ females with *tll^1^*/TM3 *Sb* males. Embryos from this cross were compared to embryos derived from *bks^278^*/+ females crossed to wt males, and with embryos derived from the *tll^1^* stock.

### Chromatin immunoprecipitation and real-time PCR.

A detailed description of this procedure can be found in Protocol S1. In brief, we established a stable S2 cell line expressing V5-tagged Bks-B, prepared sheared chromatin from this and a control S2 cell line, as well as from 2–4-h wt embryos, and performed ChIP essentially according to the Upstate ChIP assay kit protocol (Upstate Biotechnology, http://www.upstate.com). Real-time PCR was performed on an ABI prism 7000 machine using Power SYBR Green reagent (Applied Biosystems, http://www.appliedbiosystems.com). PCR was performed on 1 μl (cells) or 3 μl (embryos) template DNA in triplicate samples, and immunoprecipitated DNA was compared against standard curves from serial dilutions of input DNA. The values are plotted as percent input DNA from the corresponding extract, and the standard deviation within the triplicate samples indicated. Similar results were obtained in independent ChIP experiments.

## Supporting Information

Figure S1 Protein Sequence Alignment of Brakeless Homologs The sequences spanning the D2 domain and the C_2_H_2_ zinc finger were aligned with ClustalW (DNASTAR Lasergene, http://www.dnastar.com). Species included in the analysis are the fruit flies Drosophila melanogaster (Dm) and D. pseudoobscura (Dp), the mosquitoes Anopheles gambiae (Ag) and Aedes aegypti (Aae), honeybee Apis mellifera (Am), flour beetle Tribolium casteneum (Tc), sea urchin Strongylocentrotus purpuratus (Sp), pufferfish Tetraodon nigroviridis (Tn), zebrafish Danio rerio (Dr), mouse Mus musculus (Mm), and human Homo sapiens (Hs). In vertebrates, two Brakeless homologs, ZNF608 and ZNF609, are present. The zebrafish ZNF608 sequence is not full length.
(3.9 MB TIF)

Figure S2 Expression of Gap Gene Regulators Is Uncompromised in *bks* Mutant Embryos Embryos derived from Oregon-R (wt) or *bks^14^* germline clones *(bks)* are oriented with anterior to the left and dorsal up.
(A and B) Embryos were hybridized with a *tailless (tll)* probe. More than 90% of *bks* embryos show a *tll* pattern indistinguishable from wt (compare [B] with [A]). In a small fraction of *bks* embryos, the posterior *tll* pattern expands towards the anterior (unpublished data).
(C and D) Incubation of wt (C) and *bks* mutant (D) embryos with a *hunchback (hb)* RNA probe reveals no difference in staining pattern.
(E and F) Staining of wt (E) and *bks* mutant (F) embryos with a Hunchback (Hb) antibody demonstrates absence of Hunchback protein from the posterior in both genotypes.
(G and H) The Caudal (Cad) protein gradient extends to a similar position in *bks* mutant embryos (H) as in wt (G).
(6.1 MB TIF)

Protocol S1 Supplemental Methods (83 KB DOC)

Table S1 *kni* Repression by Misexpressed Tll (34 KB DOC)

### Accession Numbers

The GenBank (http://www.ncbi.nlm.nih.gov/Genbank) accession number for the Drosophila melanogaster Bks-B cDNA is AF242194.

## Abbreviations

aa: amino acid
bp: base pair
CD: central domain
ChIP: chromatin immunoprecipitation
CRM: *cis*-regulatory DNA module
EL: egg length
GFP: green fluorescent protein
kb: kilobase
NEE: neuroectoderm enhancer
RT-PCR: reverse transcription PCR
wt: wild-type
