# Supplementary material for: Drosophila Brakeless Interacts with Atrophin and Is Required for Tailless-Mediated Transcriptional Repression in Early Embryos
Source: PLoS Biol. 2007 May 15;5(6):e145. doi: 10.1371/journal.pbio.0050145 (PMC1868043; doi:10.1371/journal.pbio.0050145)
Supplement: Supplementary file 1 [file pbio.0050145.sd001.doc]

## Haecker et al. Protocol S1

**Fly stocks and generation of germline clones**

*Drosophila melanogaster* stocks were maintained at 18°C, 25°C or room temperature. *Oregon-R* or *w^1118^* were used as wild-type controls. *tll^1^*/TM3 Sb (hypomorph, C34Y amino acid replacement) was obtained from the Bloomington stock center (stock #2729). Stocks #1929 and 2125 used for the FLP-FRT dominant female sterile technique to create germline clones are described in (Chou and Perrimon 1996). *bks* mutant females were crossed with males of the genotype hs-FLP/Y; FRT^2R-G13^ ovo^D1^/CyO, and larvae heatshocked for 3 hr at 37^O^C on days 3, 4 and 5 after egglaying to induce expression of the FLP recombinase. Cy^+^ females were mated with *bks* heterozygous males, with wild-type males or with males expressing various transgenes, and embryos collected and fixed for *in situ* hybridization.

**Molecular cloning and P-element transformation**

The expressed sequence tag (EST) clone LD13770 represents a full length *bksA* cDNA clone. A *bksB* cDNA was assembled from ESTs LD13770 and SD01229, which covers a 3’ 7.2kb fragment of bks-B, by use of a common and unique *Not*I site (see (Senti et al. 2000)). A *Kpn*I restriction site was introduced upstream of the ATG start codon by amplifying LD13770 with primers bks-A-KpnI-L: ctagTCTAGAcgGGTACCggaATGGAAAAGAAGTTGGCA (*Xba*I and *Kpn*I sites underlined) and bks-A-KpnI-R: CTCAATATCCGCGTCCAAGT. This PCR product was digested with *Xba*I and *Xho*I (that cuts at an internal *Xho*I site) and cloned into LD13770 that was first cut with *Xba*I (in the pBS polylinker) and then partially digested with *Xho*I. This replaces the 528 bp *Xba*I-*Xho*I sequence that includes the 5’ UTR, with the 370 bp PCR product, producing the BksA-Kpn plasmid. The PCR amplified part was sequenced to verify that no mutations were introduced during PCR. The BksA-Kpn plasmid was fused with the Bks-B 3’ part from SD01229 by using the unique *Not*I site to produce BksB-Kpn. To test the Bks proteins for repressor activity**,** BksA and BksB coding sequences were fused in frame to the Tetracycline Repressor DNA binding domain (TetR) in the pAct-TetR cell culture vector (described in (Ryu and Arnosti 2003)) and to the GAL4 DNA binding domain in the pKreggy P-element transformation vector (described in (Nibu et al. 1998) where it is called KREG). A *Kpn*I fragment from BksA-Kpn was cloned into the *Kpn*I site of pAct-TetR to create TetR-BksA, and a *Kpn*I-*Spe*I fragment from BksB-Kpn was cloned into pAct-TetR cut with *Kpn*I and *Xba*I to generate TetR-BksB. The *Kpn*I fragment from BksA-Kpn was cloned into the *Kpn*I site of the pKreggy vector. BksB-V5 was created by first cloning a *Kpn*I fragment from BksB-Kpn into the pAc5.1/V5-His A vector (Invitrogen) containing the actin promoter, generating pAct-BksB. The 3’ end of bks was replaced with a PCR product that changes the stop codon and introduces an *Xba*I site (actagTCTAGATTTCGACGGACCCGGCGGAAAC). The forward primer used was TCAGCCGGACCGTATAATAC. The PCR product, digested at an internal *Rsr*II site and with *Xba*I, was cloned into the *Rsr*II and *Xba*I sites of pAct-BksB to produce pAct-BksB-V5.

GST-BksA was produced by digesting the BksA-Kpn plasmid with *Kpn*I and ligating the Bks fragment into a modified pGEX vector carrying an in frame *Kpn*I site. Digestion of pGEX-BksA with *Not*I followed by re-ligation generated a GST-Bks-1-780 fusion protein. GST-Bks-835-1151 was made by PCR amplification using primers fwd: ctagataGAATTCacgacgataccaaatctccg (*EcoR*I site underlined) and rev: ctagataCTCGAGcatggagtcctcatccatgga (containing a *Xho*I site) and ligating the digested PCR product into the pGEX 5X-2 vector. GST-ZNF608 (aa1-600) was produced by PCR amplification from the human cDNA clone KIAA1281 with primers fwd: ctagTCTAGAatgtcagtgaacatttctactg and reverse: ctagGCGGCCGCttcctcacagtccgagatcttg. The PCR product and the pGEX-5X-3/KX vector was digested with *Xba*I and *Not*I and ligated to create an in-frame GST-ZNF608-aa1-600 fusion protein.

To make the CasPeR-sna-tll transgene, a 2.6-kb *Xho*I-*Xba*I fragment from a pBS-tll subclone was blunt-ended and ligated into the *Pme*I site of CaSpeR-sna-FRT (Andrioli et al. 2002). This fragment contains the *tll* coding region followed by the *even-skipped* 3' UTR. Full length *tll* coding sequence was PCR amplified using primers fwd: CACCATGCAGTCGTCGGAGGGTTC and reverse: GATCTTGCGCTGACTGTACATGTCG. It was then cloned into the pENTR/D-TOPO vector using the TOPO cloning system (Invitrogen). It was recombined into pAWF (Terence Murphy, Carnegie Institution of Washington) and pDEST15 (Invitrogen) using the Gateway system to generate FLAG-Tll driven by the actin5C promoter and GST-Tll. Amino acids 101-452 in Tll were PCR amplified using Pfu polymerase and primers fwd: gcgcGGATCCatgaacaaggatgca (*BamH*I site underlined) and rev: gcgcCTCGAGtcagatcttgcgctgact (*Xho*I site underlined). The PCR product was digested and ligated into pGEX-4T-1.

Amino acids 1324-1966 in Atrophin were PCR amplified from Atrophin cDNA (kind gift of Bernard Charroux, IBDML, Marseille) using Expand High Fidelity enzyme (Roche) and primers fwd: caacatgATCTCGAGCAGTGGAGGAGG, rev: TTATCTGGGCCGTTGTCTAAAG. The PCR product was ligated into pGEM-T Easy (Promega). The sequence corresponding to human Atrophin-1 amino acids 600-1191 was PCR amplified from the cDNA clone BC051795 (Open Biosystems) using primers fwd: TACCCTTTCCCACCGGTGCCTAC and reverse: CTACAGTGGCTTGTCGCTTTCC and ligated into the pGEM-T Easy vector.

Kreggy-BksA and CasPeR-sna-tll were injected into *w^1118^* embryos using standard procedures (Rubin and Spradling 1982).

**GST pulldown assays**

GST-fusions were expressed in *E. coli* BL21 and purified on glutathione sepharose beads. ^35^S-labeled proteins were *in vitro* translated with the TnT coupled reticulocyte lysate system (Promega), and precleared with GSH-sepharose. They were then incubated with GST-fusion proteins on GSH sepharose in NETN buffer (20mM Tris pH 8.0, 100mM NaCl, 1mM EDTA, 0.5% NP-40) for 1h. Sepharose beads were collected by a brief centrifugation at 2000rpm and washed 4 times in NETN before elution by boiling in loading buffer. Proteins were separated on a 10% polyacrylamide-SDS gel and exposed to a phosphoimager (FLA-3000, Fujifilm).

**Cuticle preparations, *in situ* hybridization and immunohistochemistry:**

*Bks* germline clone embryos were aged, dechorionated in bleach, transferred to a microscopic slide, cleared in lactic acid or Hoyer’s mountant at 65^O^C, and cuticles examined using dark-field microscopy (Wieschaus and Nüsslein-Volhard 1998). RNA *in situ* hybridization using digoxigenin-labeled probes was performed as previously described (Tautz and Pfeifle 1989; Jiang et al. 1991). Fluorescent *in situ* hybridization using a Tyramide Signal Amplification kit (cat# NEL753, PerkinElmer) was combined with conventional *in situ* hybridization. Fixed embryos were washed in ethanol and then in methanol, incubated in 2.25% H_2_O_2_ to quench endogenous peroxidase, followed by three washes in methanol. Embryos were then processed as described (Tautz and Pfeifle 1989; Jiang et al. 1991), except that fixation after proteinase K treatment was in 4% formaldehyde for 10 min. Embryos were hybridized with a fluorescein-labeled *tll* probe and with a digoxigenin-labeled *kni* probe at 55^O^C over night. After washes in hybridization buffer and PBT (PBS, 0.1% Tween 20), embryos were incubated with both peroxidase-conjugated anti-fluorescein (1:10 000) and alkaline phosphatase (AP)-conjugated anti-digoxigenin (1:200) antibodies. The embryos were incubated in 4µl Cy3-labeled tyramide in 200µl dilution buffer for 8 min, washed in PBT followed by development of the AP-catalyzed reaction. After washing in PBT, the embryos were dehydrated in ethanol and mounted in Permount (Fisher).

The immunohistochemistry protocol is modified from (Manoukian and Krause 1992). Embryos were fixed in 4% formaldehyde and devitellinized in methanol. Then, they were rehydrated in PBTH buffer (1x PBS, 0,1% Tween 20, 250 ug/ml Heparin) for 1h. The primary antibody was added and incubated overnight at 4°C. The mouse anti-BKS antibody (1:50 dilution, directed against the conserved D1 region) was kindly provided by Barry Dickson and is described in (Senti et al. 2000). The guinea pig anti-CAD antibody (1:800 dilution) is a gift from John Reinitz and is described in (Kosman et al. 1998). The rat anti-HB antibody (1:400 dilution, described in (Kosman et al. 1998)) was provided by Steve Small. Embryos were washed in PBTH and the CAD antibody was detected with PAN Antibody using the Vectastain ABC Elite kit. The HB- and BKS-antibodies were detected with Cy3-labeled anti-rat or anti-mouse antibodies (Jackson Laboratories).

**RT-PCR**

Total RNA was isolated from *w^1118^* (wild-type) embryos (collected 0-3h after egg laying) by use of TRIzol reagent (Invitrogen). Five micrograms of total RNA was reverse transcribed with Superscript II RNAse H-free reverse transcriptase (RT) (Invitrogen) using an oligo-dT primer. Specific primers to distinguish between Bks-A and Bks-B were used in PCR on the resulting cDNA. Bks-A-L:acgacgataccaaatctccg Bks-A-R: actcaattcccaagccacac Bks-B-L: cctcctacacagcagcaaca Bks-B-R: caaatccaggcgatgtcttt. To ensure absence of genomic DNA, PCR was performed on a mock reverse transcribed RNA sample.

**Cell culture and transient transfection**

*Drosophila* S2 and mbn2 cells were grown in Schneider *Drosophila* medium (Gibco) containing 10% fetal calf serum, 1XGlutamax (Gibco), 0.05 mg/ml Gentamycin, 50 IU/ml Penicillin and 50 µg/ml Streptomycin. Cells were transfected using the Calcium Phosphate Transfection kit (Invitrogen) essentially as previously described (Ryu and Arnosti 2003). 3 x 10^6^ cells were transfected with 0.5μg of the luciferase reporter construct pAc2T, which is constitutively activated by the *Actin5C* enhancer and contains two Tet operator sites (Ryu and Arnosti 2003), with 0.1μg of the internal control pAc/lacZ and with either 0.25 μg pAct-TetR repressor, 0.25μg pTetR-bksA or 0.25μg pTetR-bksB fusion protein constructs respectively. pBS was used to equalize the total amount of DNA transfected. Cells were washed and harvested in 0.1M Tris pH 7.8 and lysed by three freeze-thaw cycles. β-galactosidase activity was measured to quantify transfection efficiency and the luciferase data were adjusted accordingly. The ß-gal assay used 225 µl of Z-buffer (0.1M sodium phosphate pH 7.0, 10mM KCl, 1mM MgSO_4_, to which 0.2% ß-mercaptoethanol was added before use) that was mixed with 25 µl of cell extract and 50 µl of *o*-nitrophenyl-*ß*-D-galactopyranoside (ONPG, 4 mg/ml 0.1M sodium phosphate pH 7.5). Absorbance at 420 nm was measured at different time points and values within the linear range (OD values 0.2 – 0.8) were used for calculations. Luciferase assays were performed following the manufacturer’s instructions (Promega) and quantified using a Luminescent Image Analyzer (LAS-1000plus, Fujifilm). The corrected luciferase values plotted in Fig. 5 are derived from three independent experiments.

**Immunoprecipitation**

We established a stable cell line expressing V5-tagged Bks-B by co-transfecting 5 ug of pAct-BksB-V5 with 0.25 ug pCoBlast plasmid into *Drosophila* S2 cells by calcium phosphate transfection. Cells were selected in Schneider *Drosophila* medium containing 50 ug/ml blasticidin and maintained in medium containing 10 ug/ml blasticidin. Immunostaining with V5 antibody (Invitrogen) showed that approximately 10% of the cells expressed BksB-V5. Two ug FLAG-tagged Tll was transiently transfected into the BksB-V5 cell line. Immunoprecipitation was performed as previously described (Qi et al. 2006). 1 ul V5 monoclonal antibody (Invitrogen), 3 ul Atrophin guinea-pig polyclonal antibody (a gift from Chih-Cheng Tsai, UMDNJ-Robert Wood Johnson Medical school) and 5 ul of an anti-FLAG rabbit antibody (Sigma) were used for immunoprecipitation from transfected and untransfected cells. The protein membrane was probed sequentially with the V5 antibody and a FLAG M2 monoclonal antibody (Sigma).

### Chromatin immunoprecipitation and real-time PCR

Forty ml of BksB-V5 expressing cells and 40 ml of control S2 cells (5x10^6^ cells/ml) were cross-linked with 1% formaldehyde for 10 min at room temperature, and the reaction stopped by addition of 2.5 ml 1.25M glycine pH 7.0. The cells were sequentially washed with ice-cold PBS, ChIP wash buffer A (10mM Hepes pH 7.9, 10mM EDTA, 0.5 mM EGTA, 0.25% Triton X-100), and ChIP wash buffer B (10mM Hepes pH 7.9, 100mM NaCl, 1mM EDTA, 0.5 mM EGTA, 0.01% Triton X-100). Sonication was performed in 5 ml TEN140 (10mM Tris pH 8.0, 0.1 mM EDTA, 140 mM NaCl) containing 100 ug/ml PMSF. The size of the DNA fragments after sonication was between 200 and 800 bp. After sonication, the lysate was adjusted to RIPA buffer (0.1% SDS, 0.1% deoxycholate, 1% Triton X-100) and centrifuged. The supernatant was aliquoted into eppendorf tubes (150 ul/tube) and quick frozen in liquid nitrogen. One tube (150 ul) corresponding to 5x10^6^ cells was used for each immunoprecipitation

Two-four hours wt embryos were collected, dechorionated and washed with water and NaCl-Triton buffer. Every 100ul of embryos were fixed in one 2ml eppendorf tube containing 0.8ml heptane and 0.8ml 2% PFA in PBS for 25 minutes with shaking. The vitteline membrane was removed by shaking vigorously in methanol. The embryos were washed three times with methanol and twice with ice-cold storage buffer (50mM Tris-HCl pH8.0; 1mM EDTA) and kept at -80°C until 1ml of embryos were obtained. Embryos were then washed once with 10 volumes of SDS-lysis buffer (100mM NaCl; 50mM Tris-HCl pH8.0; 5mM EDTA; 0.5% SDS) and suspended in 10 volumes IP buffer (6.6 volumes of SDS-Lysis buffer and 3.3 volumes Triton buffer (100mM NaCl; 100mM Tris-HCl pH8.0; 5mM EDTA; 5% Triton X-100)) containing protease inhibitors. Sonication was performed in 1.5ml eppendorf tubes containing 100ul of embryos and 1 ml buffer. The lysate was spun at 4°C (maximum speed), the supernatants pooled, and 900ul of the resulting extract used for one ChIP reaction.

Chromatin immunoprecipitation (ChIP) was done according to the Upstate ChIP assay kit protocol with a few modifications. The extract was diluted and precleared with 10% Normal Rabbit Serum overnight at 4^0^C followed by addition of 30 ul Dynabeads protein A + protein G magnetic beads (Dynal, coated with salmon sperm DNA and BSA) for one hour. It was then incubated with 3 ul V5 (Invitrogen), 3 ul GFP (Sigma), 10 ul affinity purified Bks, 3 ul Atrophin (from C-C. Tsai) or 3 ul rabbit anti-Tll (Kosman et al. 1998) antibody overnight at 4^o^C. Thirty ul coated protein A/G beads were added for one hour, and the immuncomplexes washed, eluted and the cross-links reversed. DNA was recovered by phenol/chloroform extraction and ethanol precipitation and resuspended in 60 ul (cell extract) or 20 ul (embryo extract) double distilled H_2_O.

Real-time PCR was performed on an ABI prism 7000 machine using Power SYBR Green reagent (Applied Biosystems). Primers were designed with Primer Express software (Applied Biosystems), and the primer concentration optimized to avoid primer dimers. The primer pairs are located in the 3’ half of the 1 kb *kni* CRM, at position 32-132 in the *kni* cDNA, in the 3’ end of the 730 bp *Kr* CRM, and 2.7 kb into the transcript of gene CG31998 on chromosome 4 (sequences available upon request). PCR was performed on 1 ul (cells) or 3 ul (embryos) template DNA in triplicate samples and immunoprecipitated DNA was compared against standard curves from serial dilutions of input DNA. The values are plotted as % input DNA from the corresponding extract, and the standard deviation within the triplicate samples indicated. Similar results were obtained in independent ChIP experiments.

# Bks antibody production

A rat anti-Bks serum was rasied against the N-terminus of Bks. His-Bks-aa450-620 was produced by PCR amplification using primers fwd: actgGAATTCaAAGATGTCCATAGACCACC and rev: actgAAGCTTaGGCAGCAGCTGCTGACTTG and cloned into the pRSET expression vector using *EcoR*I and *Hind*III restriction sites. GST-Bks-aa450-620 was produced by PCR amplification using an alternative rev primer with a *Xho* I site instead of the *Hind*III site and cloned into the pGEX-5X vector.

The His-tagged protein was expressed in *E. coli* and injected into rat. The serum was then affinity purified by passing over GST-Bks450-620 coupled to Affi-Gel 10 (Bio-Rad). The Affi-gel column was washed and the antibody eluted by low pH into 2M Tris pH8.0.

# References

Andrioli, L.P., V. Vasisht, E. Theodosopoulou, A. Oberstein, and S. Small. 2002. Anterior repression of a Drosophila stripe enhancer requires three position-specific mechanisms. *Development* **129**: 4931-40.

Chou, T.B. and N. Perrimon. 1996. The autosomal FLP-DFS technique for generating germline mosaics in Drosophila melanogaster. *Genetics* **144**: 1673-9.

Jiang, J., T. Hoey, and M. Levine. 1991. Autoregulation of a segmentation gene in Drosophila: combinatorial interaction of the even-skipped homeo box protein with a distal enhancer element. *Genes Dev* **5**: 265-77.

Kosman, D., S. Small, and J. Reinitz. 1998. Rapid preparation of a panel of polyclonal antibodies to Drosophila segmentation proteins. *Dev Genes Evol* **208**: 290-4.

Manoukian, A.S. and H.M. Krause. 1992. Concentration-dependent activities of the even-skipped protein in Drosophila embryos. *Genes Dev* **6**: 1740-51.

Nibu, Y., H. Zhang, E. Bajor, S. Barolo, S. Small, and M. Levine. 1998. dCtBP mediates transcriptional repression by Knirps, Kruppel and Snail in the Drosophila embryo. *Embo J* **17**: 7009-20.

Rubin, G.M. and A.C. Spradling. 1982. Genetic transformation of Drosophila with transposable element vectors. *Science* **218**: 348-53.

Ryu, J.R. and D.N. Arnosti. 2003. Functional similarity of Knirps CtBP-dependent and CtBP-independent transcriptional repressor activities. *Nucleic Acids Res* **31**: 4654-62.

Senti, K., K. Keleman, F. Eisenhaber, and B.J. Dickson. 2000. brakeless is required for lamina targeting of R1-R6 axons in the Drosophila visual system. *Development* **127**: 2291-301.

Tautz, D. and C. Pfeifle. 1989. A non-radioactive in situ hybridization method for the localization of specific RNAs in Drosophila embryos reveals translational control of the segmentation gene hunchback. *Chromosoma* **98**: 81-5.

Wieschaus, E. and C. Nüsslein-Volhard. 1998. Looking at embryos. In *Drosophila, a practical approach* (ed. D.B. Roberts), pp. 197-201. IRL Press, Oxford.
