# Supplementary material for: Drosophila Brakeless Interacts with Atrophin and Is Required for Tailless-Mediated Transcriptional Repression in Early Embryos
Source: PLoS Biol. 2007 May 15;5(6):e145. doi: 10.1371/journal.pbio.0050145 (PMC1868043; doi:10.1371/journal.pbio.0050145)
Supplement: Supplementary file 2 [file pbio.0050145.st001.doc]

**Supplemental Table S1**. *kni* repression by mis-expressed Tll

| *sna:tll* transgene | *kni* repression (%) | Embryos (n) |
| --- | --- | --- |
| X196 in wt | 24.0 | 312 |
| X196 in *bks^278^* | 8.8 | 148 |
| X197 in wt | 38.5 | 179 |
| X197 in *bks^278^* | 14.7 | 61 |

| *sna:tll* transgene | *kni* repression (%) | Embryos with transgene (n) |
| --- | --- | --- |
| X196 in wt | 60.0 | 30 |
| X196 in *bks^278^* | 26.3 | 19 |
| X197 in wt | 74.2 | 31 |
| X197 in *bks^278^* | 0 | 7 |

Two transgenic *sna:tll* lines (X196 and X197) were crossed to wild-type (wt) females or to females with *bks^278^* germline clones, embryos collected and stained for *kni* expression. In the lower panel, embryos were stained with both *kni* and *tll* probes, and only embryos where mis-expressed *tll* could be detected analyzed. All differences between wt and *bks* mutants are statistically significant (at least p<0.05) as determined by pair-wise chi^2^ tests.
